# Supplementary material for: Mutations to the HCoV-229E spike have counterbalancing effects on serum antibody neutralization and receptor binding
Source: bioRxiv. 2026 Feb 23:2026.02.22.707297. Preprint. [Version 1] doi: 10.64898/2026.02.22.707297 (PMC13160040; doi:10.64898/2026.02.22.707297)
Supplement: Supplement 2 [file NIHPP2026.02.22.707297v1-supplement-2.pdf]

bioRxiv preprint doi: https://doi.org/10.64898/2026.02.22.707297; this version posted February 23, 2026. The copyright holder for this preprint (which was not certified by peer review) is the author/funder, who has granted bioRxiv a license to display the preprint in perpetuity. It is made available under aCC-BY 4.0 International license.

Supplementary Tables

Supplementary Table S1. Amino acid mutations in HCoV-229E spike proteins used to test the neutralization resistance or sensitivity of the human sera relative to the 1984 strain used in the deep mutational scanning.

229E paper-supp table 1

bioRxiv preprint doi: https://doi.org/10.64898/2026.02.22.707297; this version posted February 23, 2026. The copyright holder for this preprint (which was not certified by peer review) is the author/funder, who has granted bioRxiv a license to display the preprint in perpetuity. It is made available under aCC-BY 4.0 International license.

Supplementary Figures

bioRxiv preprint doi: https://doi.org/10.64898/2026.02.22.707297; this version posted February 23, 2026. The copyright holder for this preprint (which was not certified by peer review) is the author/funder, who has granted bioRxiv a license to display the preprint in perpetuity. It is made available under aCC-BY 4.0 International license.

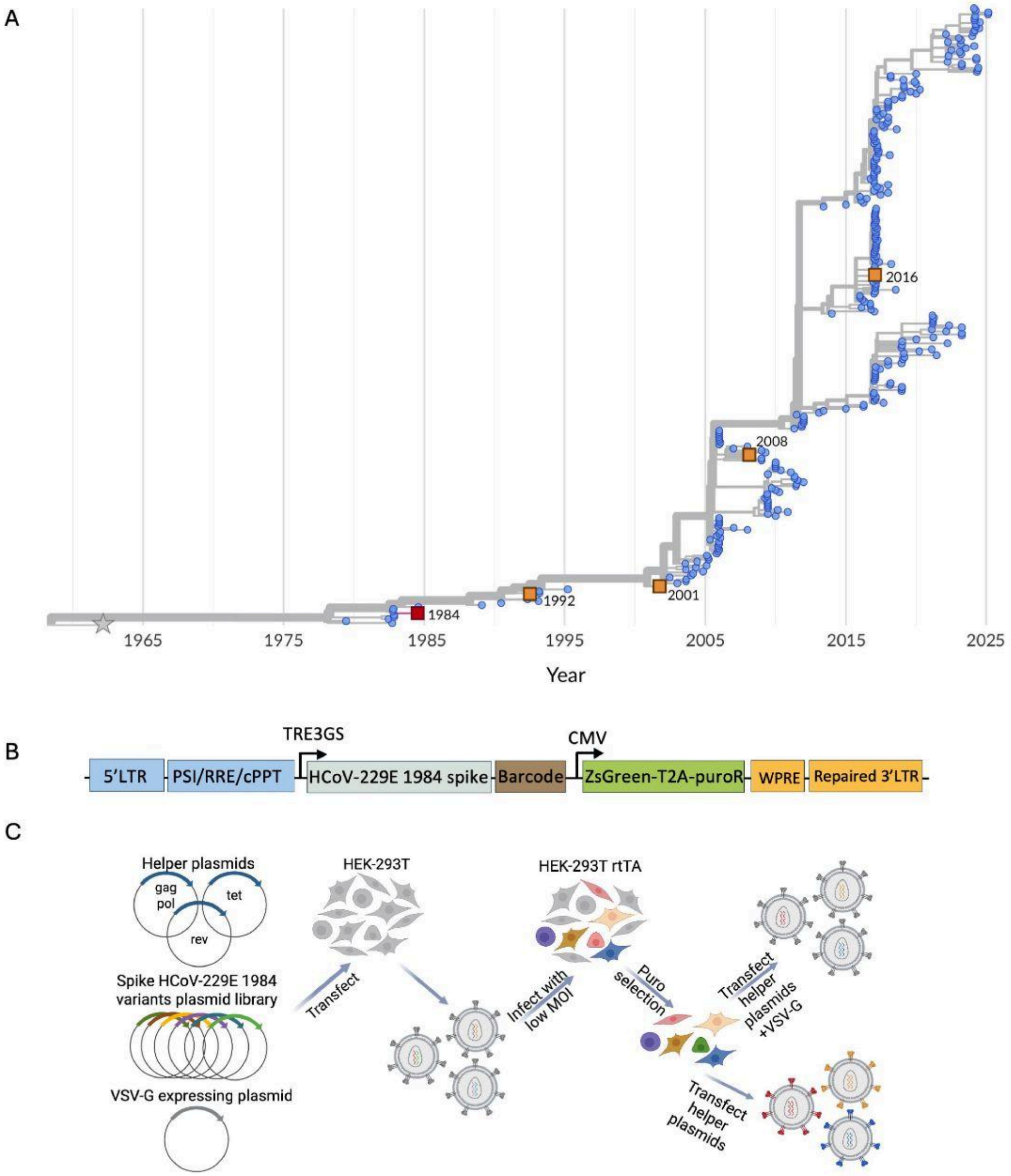

D

Library	# of designed mutations	# of AA mutations	% of maximum AA mutation	# of barcodes
Library A	21,301	20,480	96.1	91,028
Library B	21,301	20,742	97.3	96,910

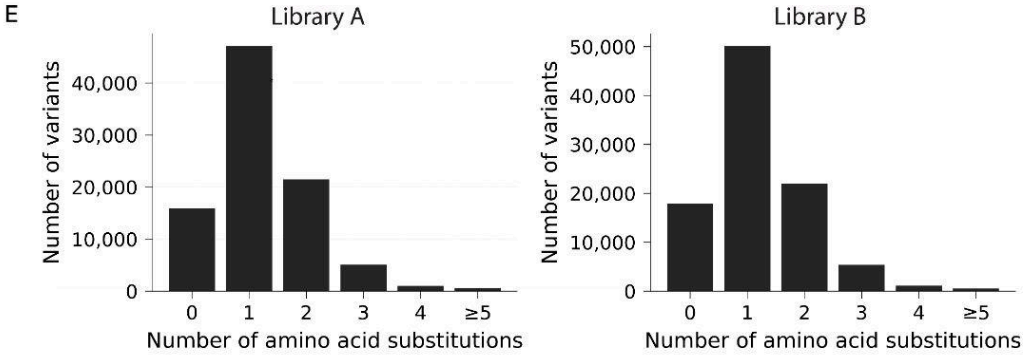

bioRxiv preprint doi: https://doi.org/10.64898/2026.02.22.707297; this version posted February 23, 2026. The copyright holder for this preprint (which was not certified by peer review) is the author/funder, who has granted bioRxiv a license to display the preprint in perpetuity. It is made available under aCC-BY 4.0 International license.

Supplementary Figure S1. Pseudotyped lentivirus spike libraries design and production.

(A) Time-scaled phylogeny of the HCoV-229E spike inferred using the full spike nucleotide sequences. Red square marks the HCoV-229E 1984 spike that is used for this study. Additional strains used in neutralization assays to identify evolution-sensitive versus evolution-resistant sera are marked with orange squares. The reference strain (NC_002645) from a 1962 isolate, marked as grey star, was extensively passaged in the lab prior to sequencing. See https://nextstrain.org/community/jbloomlab/cov-229E-spike-phylo@main for an interactive version of this phylogenetic tree. (B) Schematic of the lentiviral genome (backbone) used for pseudovirus deep mutational scanning[143]. The backbone contains standard lentiviral elements (5′ LTR, packaging signal Ψ, RRE, cPPT, and 3′ LTR), with a full-length (non-deleted) 3′ LTR to enable reactivation of integrated proviruses. It encodes the HCoV-229E spike protein under an inducible TRE3GS promoter, followed by a random nucleotide barcode placed downstream of the stop codon. A constitutive CMV promoter drives expression of ZsGreen and a puromycin-resistance cassette. (C) Workflow to generate a library of pseudotyped lentiviral particles. 293T cells are transfected with the library of plasmids encoding the spike variants together with lentiviral helper plasmids (encoding Tat, Gag-Pol, and Rev), and a plasmid expressing VSV-G. The resulting virions are used to infect 293T cells expressing rTTA at low multiplicity of infection (0.01). Infected cells with an integrated lentiviral genome are selected using puromycin; due to the low multiplicity of infection nearly all of these cells contain just a single integrated genome encoding a barcoded spike variant. The genotype-phenotype linked lentiviral library is then produced by retransfecting these cells with the helper plasmids. To quantify library composition independent of the spike function, particles pseudotyped with VSV-G are also generated and used as a control to normalize library composition independent of spike function. This panel was created with BioRender.com (D) Number of designed (intended) unique amino-acid mutations to be covered in each library, actual number of unique mutations represented at least once in each library, and number of uniquely barcoded spike variants in each library. (E) Distribution of the number of amino-acid mutations per barcoded variant for each library.

bioRxiv preprint doi: https://doi.org/10.64898/2026.02.22.707297; this version posted February 23, 2026. The copyright holder for this preprint (which was not certified by peer review) is the author/funder, who has granted bioRxiv a license to display the preprint in perpetuity. It is made available under aCC-BY 4.0 International license.

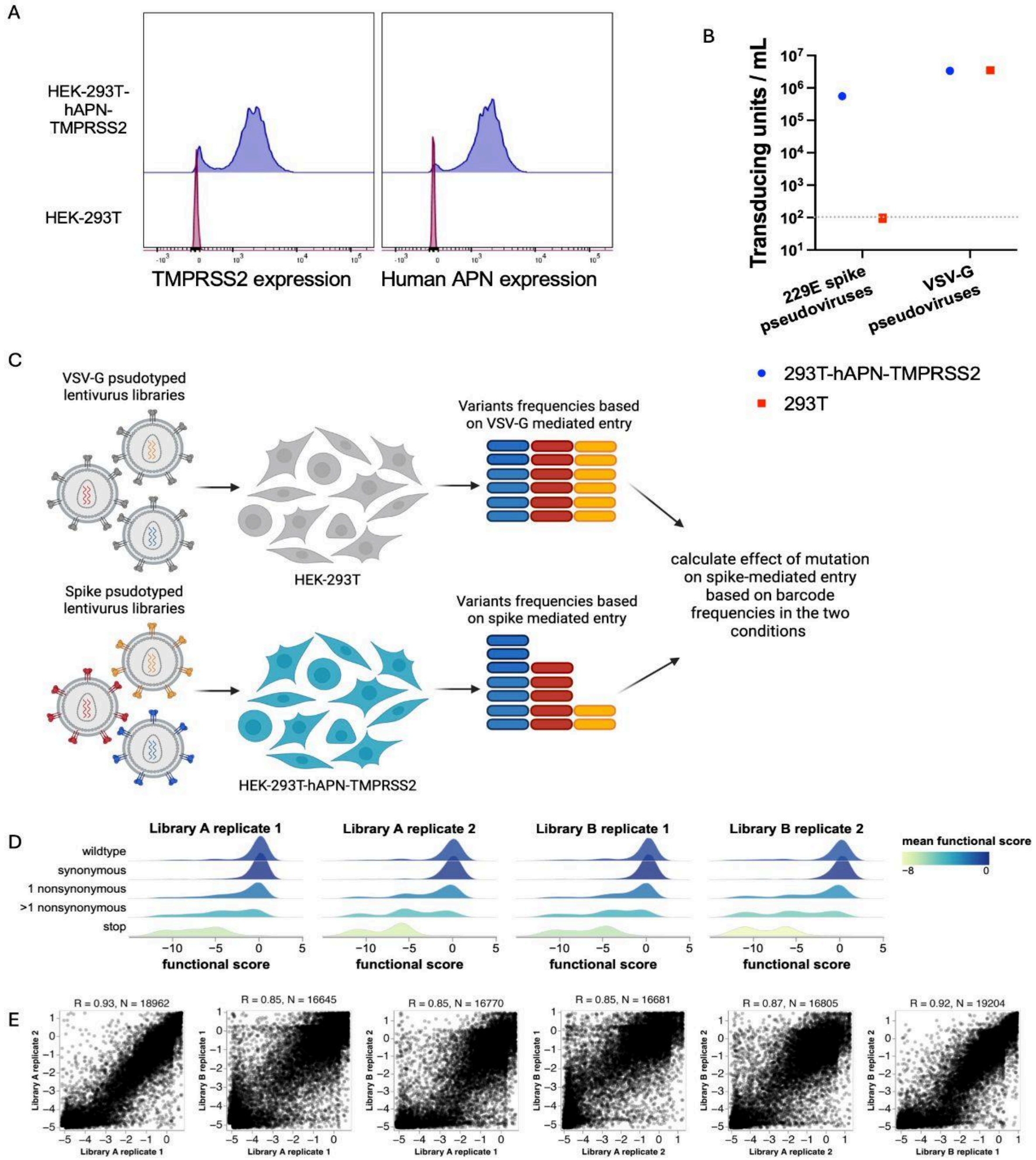

Supplementary Figure S2. Measurement of effects of spike mutations on cell entry.

(A) Expression of human amino-peptidase N (hAPN) and TMPRSS2 in the 293T-hAPN-TMPRSS2 cell clone used in this paper. Expression of hAPN was quantified by antibody staining and flow cytometry, while expression of TMPRSS2 was quantified by flow cytometry analysis of fluorescence of a mCherry reporter expressed off the same transcript. (B) Infectious titers of

bioRxiv preprint doi: https://doi.org/10.64898/2026.02.22.707297; this version posted February 23, 2026. The copyright holder for this preprint (which was not certified by peer review) is the author/funder, who has granted bioRxiv a license to display the preprint in perpetuity. It is made available under aCC-BY 4.0 International license.

lentiviral particles pseudotyped with either the 229E spike or VSV-G, measured on 293T and 293T-hAPN-TMPRSS2 target cells. As expected, spike pseudotyped viral particles can only efficiently infect the cells that express the hAPN receptor and TMPRSS2 activating protease. (C) Schematic of deep mutational scanning workflow for measuring entry into 293T-hAPN-TMPRSS2 cells. Cells are infected with either the VSV-G pseudotyped library or the spike pseudotyped variant library produced as described in Fig S1. At 12 hours post-infection, viral DNA is extracted from infected cells, and barcode frequencies are quantified by sequencing[143]. All variants can enter cells equally when pseudotyped with VSV-G, but for the spike-pseudotyped virions entry is dependent on spike function. A cell entry score is computed by comparing barcode frequencies between the spike library and the VSV-G control library conditions. This panel was created with BioRender.com (D) Distributions of cell entry scores for variants grouped by the type of mutation they contain. A score of zero corresponds to entry equivalent to the unmutated spike, while negative values indicate reduced entry. (E) Correlation of per-mutation cell entry effects measured in two technical replicate experiments across each of the two independent biological replicate libraries, after inferring mutation-level effects from variant scores using global epistasis models (Methods). Numbers above plots give the Pearson correlation (R) and number of mutations (N) measured in both pairs of replicates.

bioRxiv preprint doi: https://doi.org/10.64898/2026.02.22.707297; this version posted February 23, 2026. The copyright holder for this preprint (which was not certified by peer review) is the author/funder, who has granted bioRxiv a license to display the preprint in perpetuity. It is made available under aCC-BY 4.0 International license.

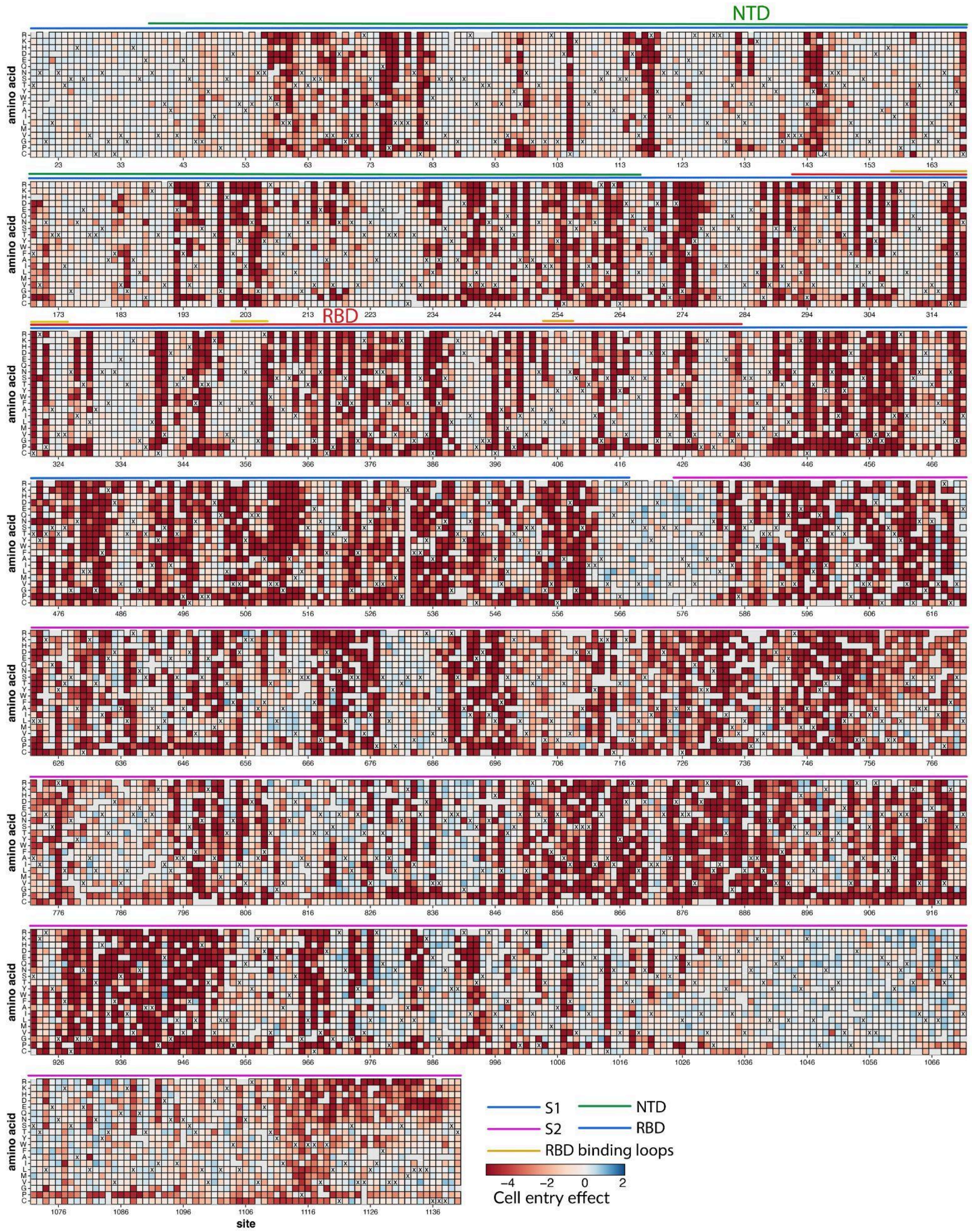

Supplementary Figure S3. Effects of mutations to the HCoV-229E spike on entry in 293T-hAPN-TMPRSS2 cells

Each square in the heatmap represents the effect of an amino-acid mutation, with red indicating impaired entry, white indicating wildtype-like entry, and blue indicating improved

bioRxiv preprint doi: https://doi.org/10.64898/2026.02.22.707297; this version posted February 23, 2026. The copyright holder for this preprint (which was not certified by peer review) is the author/funder, who has granted bioRxiv a license to display the preprint in perpetuity. It is made available under aCC-BY 4.0 International license.

entry. The wildtype amino acid of the 1984 HCoV-229E spike used for the deep mutational scanning is indicated with an "X" at each site. The handful of gray squares indicate mutations that were not measured with high confidence in the deep mutational scanning. The lines above the heatmap indicate different regions of spike. See https://dms-vep.org/229E_spike_1984_DMS/cell_entry.html for an interactive version of this heatmap.

bioRxiv preprint doi: https://doi.org/10.64898/2026.02.22.707297; this version posted February 23, 2026. The copyright holder for this preprint (which was not certified by peer review) is the author/funder, who has granted bioRxiv a license to display the preprint in perpetuity. It is made available under aCC-BY 4.0 International license.

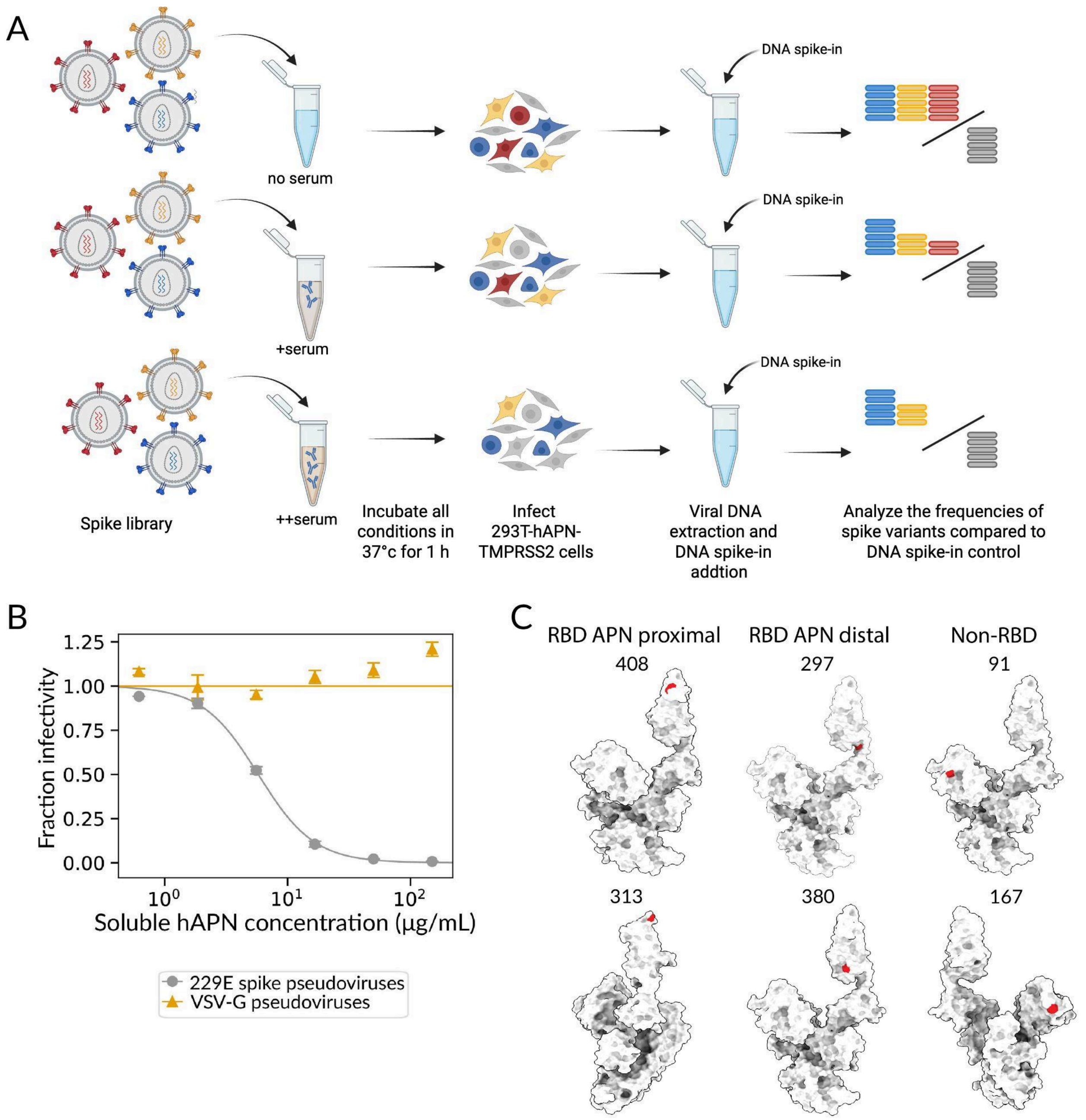

Supplementary Figure S4. Measurement of effects of spike mutations on hAPN binding.

(A) Schematic of deep mutational scanning to measure the effects of spike mutations on hAPN binding using 293T-hAPN-TMPRSS2 cells. The spike pseudotyped variant library is mixed with barcoded VSV-G pseudotyped lentiviral particles that act as a standard for normalization of sequencing counts, and incubated with increasing concentrations of soluble hAPN, including a no hAPN control. After 1h incubation at 37C°, cells are infected with the pseudovirus mix, and at 12 hours post-infection the viral DNA is extracted from infected cells, and barcode frequencies are quantified by sequencing[29]. VSV-G barcode counts are used to normalize the read counts of spike variants across conditions to determine the absolute infectivity of each spike variant at

bioRxiv preprint doi: https://doi.org/10.64898/2026.02.22.707297; this version posted February 23, 2026. The copyright holder for this preprint (which was not certified by peer review) is the author/funder, who has granted bioRxiv a license to display the preprint in perpetuity. It is made available under aCC-BY 4.0 International license.

each hAPN concentration. Mutations that decrease receptor binding are neutralized less potently by soluble hAPN, while mutations that increase binding are neutralized more potently by soluble hAPN. This panel was created with BioRender.com. (B) Neutralization of pseudoviruses expressing the unmutated spike of HCoV-229E pseudoviruses or VSV-G by soluble hAPN. (C) Sites with mutations that were chosen for additional validations, displayed on one monomer of the spike protein structure PDB 8WDE[47], segregated based on their proximity to the hAPN binding as defined in Fig 3.

bioRxiv preprint doi: https://doi.org/10.64898/2026.02.22.707297; this version posted February 23, 2026. The copyright holder for this preprint (which was not certified by peer review) is the author/funder, who has granted bioRxiv a license to display the preprint in perpetuity. It is made available under aCC-BY 4.0 International license.

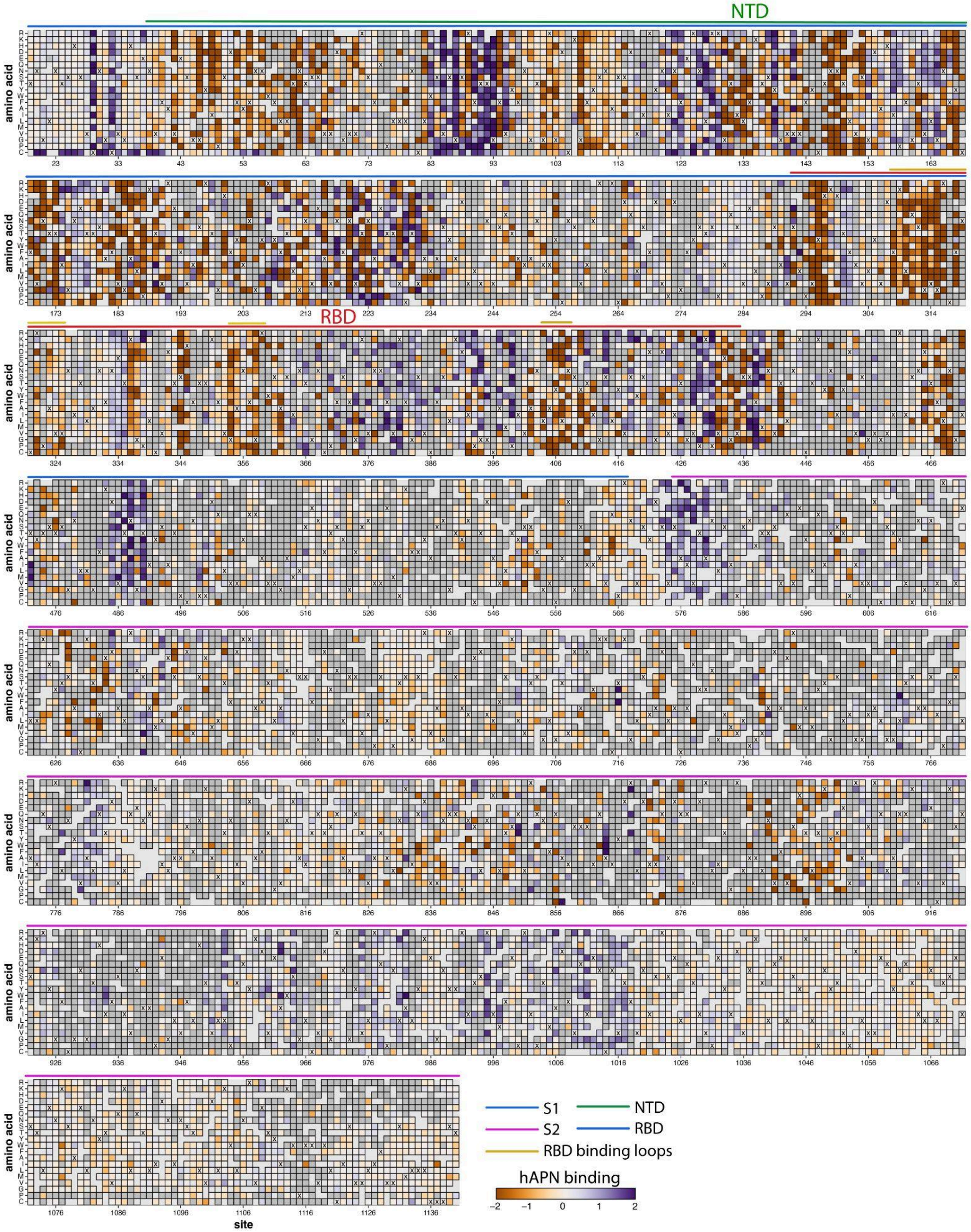

bioRxiv preprint doi: https://doi.org/10.64898/2026.02.22.707297; this version posted February 23, 2026. The copyright holder for this preprint (which was not certified by peer review) is the author/funder, who has granted bioRxiv a license to display the preprint in perpetuity. It is made available under aCC-BY 4.0 International license.

Supplementary Figure S5. Effects of mutations to the HCoV-229E spike on hAPN binding as measured by pseudovirus neutralization by soluble hAPN

Each square in the heatmap represents the effect of an amino-acid mutation, with orange indicating reduced hAPN binding, white indicating wildtype-like hAPN binding, and purple indicating improved hAPN binding. The wildtype amino acid of the 1984 HCoV-229E spike used for the deep mutational scanning is indicated with an "X" at each site. Dark gray squares are mutations that strongly impair cell entry (*cell entry effect <=−2.5*), and therefore cannot be reliably measured for their impact on receptor binding. The handful of light gray squares indicate mutations that were not measured with high confidence due to being poorly represented in the pseudovirus library. The lines above the heatmap indicate different regions of spike. See https://dms-vep.org/229E_spike_1984_DMS/APN_binding.html for interactive plots showing the effects of mutations on hAPN binding.

bioRxiv preprint doi: https://doi.org/10.64898/2026.02.22.707297; this version posted February 23, 2026. The copyright holder for this preprint (which was not certified by peer review) is the author/funder, who has granted bioRxiv a license to display the preprint in perpetuity. It is made available under aCC-BY 4.0 International license.

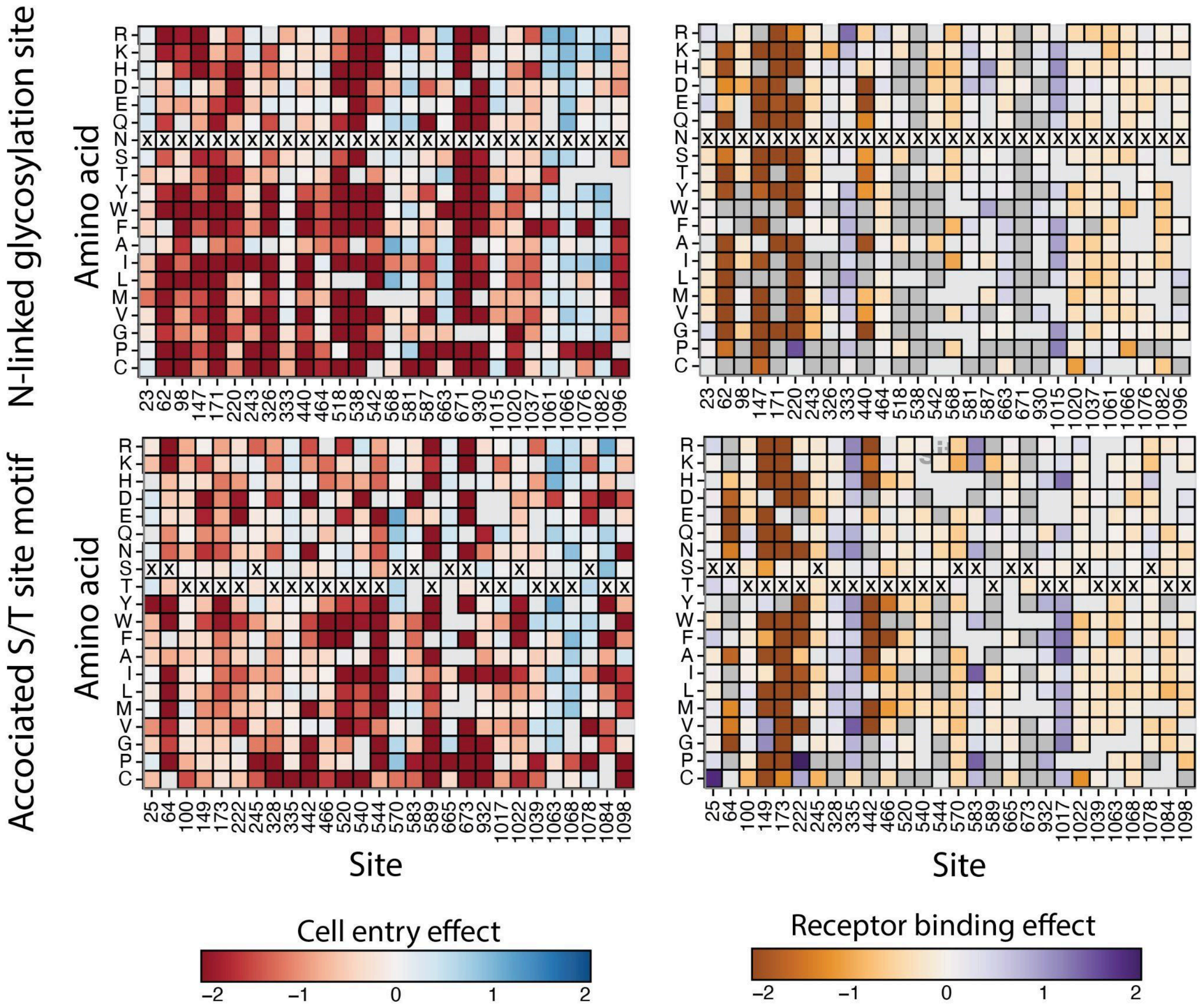

Supplementary Figure S6. Effects of mutations at N-linked glycosylation motifs on HCoV-229E spike cell entry and hAPN binding.

Heatmaps show the effects of amino-acid mutations at N-linked glycosylation motifs (N-X-S/T) and the associated motif positions on spike-mediated cell entry (left) and hAPN binding as measured by pseudovirus neutralization by soluble hAPN (right). Each square represents the effect of an amino-acid mutation, with colors indicating the direction and magnitude of the effect, as in the corresponding full heatmaps for cell entry (Fig. S3) and hAPN binding (Fig. S5). Dark gray squares indicate mutations that strongly impair cell entry (cell entry effect ≤ −2.5) and therefore cannot be reliably measured for their impact on receptor binding; light gray squares

bioRxiv preprint doi: https://doi.org/10.64898/2026.02.22.707297; this version posted February 23, 2026. The copyright holder for this preprint (which was not certified by peer review) is the author/funder, who has granted bioRxiv a license to display the preprint in perpetuity. It is made available under aCC-BY 4.0 International license.

indicate mutations that were not measured with high confidence due to poor representation in the pseudovirus library.

bioRxiv preprint doi: https://doi.org/10.64898/2026.02.22.707297; this version posted February 23, 2026. The copyright holder for this preprint (which was not certified by peer review) is the author/funder, who has granted bioRxiv a license to display the preprint in perpetuity. It is made available under aCC-BY 4.0 International license.

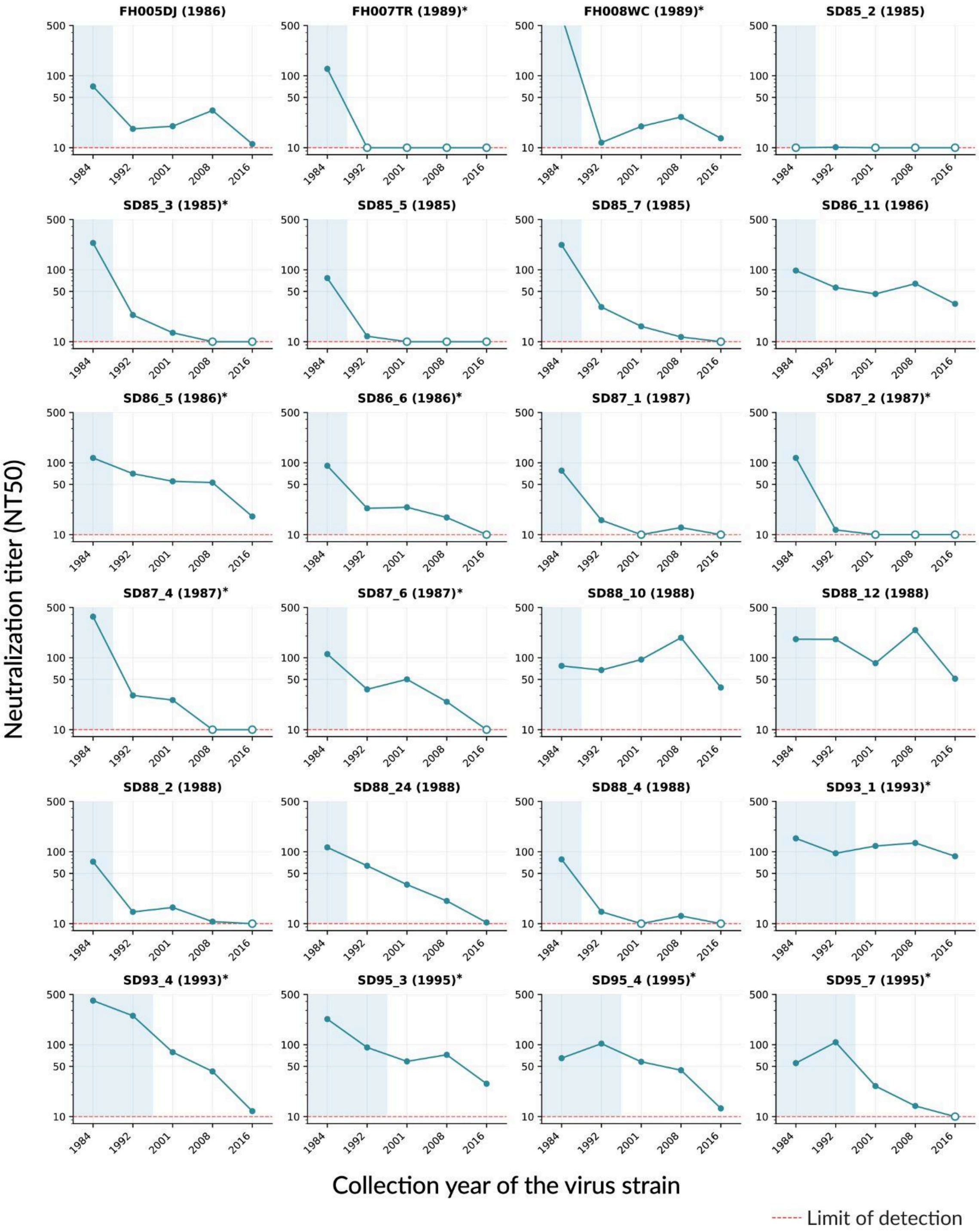

Supplementary Figure S7. Neutralization of pseudoviruses with HCoV-229E spikes from different years by all tested human sera

This plot is similar to Fig. 5 except it shows all historical human sera tested against the panel of HCoV-229E spikes from different years, whereas Fig. 5 just shows a subset of sera exemplifying either evolution-sensitive or evolution-resistant neutralization patterns.

bioRxiv preprint doi: https://doi.org/10.64898/2026.02.22.707297; this version posted February 23, 2026. The copyright holder for this preprint (which was not certified by peer review) is the author/funder, who has granted bioRxiv a license to display the preprint in perpetuity. It is made available under aCC-BY 4.0 International license.

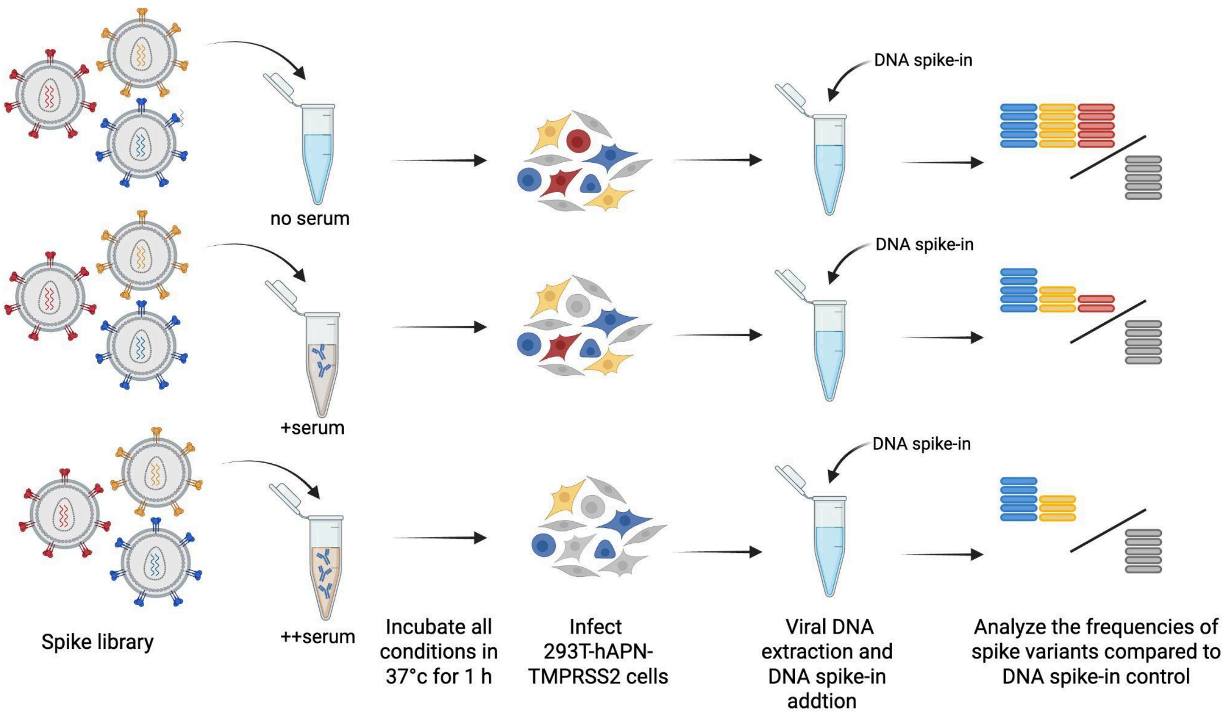

Supplementary Figure S8. Measurement of effects of spike mutations on serum neutralization escape

The spike-pseudotyped variant library is incubated with increasing concentrations of serum, including a no-serum control. After 1 h incubation at 37°C, 293T-hAPN-TMPRSS2 cells are infected with the pseudovirus mix. At 12 h post-infection, a DNA spike-in is added, and viral DNA is extracted from infected cells. Variant barcode frequencies are quantified by sequencing[29], and DNA spike-in counts are used to normalize spike-variant read counts across conditions, enabling calculation of the absolute fraction infectivity of each spike variant that is retained at each serum concentration. The fraction infectivities are analyzed to determine the effect of each mutation on serum neutralization.
